# Therapeutic Effect of Polymeric Nanomicelles Formulation of LY2157299-Galunisertib on CCl_4_-Induced Liver Fibrosis in Rats

**DOI:** 10.3390/jpm12111812

**Published:** 2022-11-01

**Authors:** Elisa Panzarini, Stefano Leporatti, Bernardetta Anna Tenuzzo, Alessandra Quarta, Nemany A. N. Hanafy, Gianluigi Giannelli, Camilla Moliterni, Diana Vardanyan, Carolina Sbarigia, Marco Fidaleo, Stefano Tacconi, Luciana Dini

**Affiliations:** 1 Department of Biological and Environmental Sciences and Technologies (Di.S.Te.B.A.), University of Salento, 73100 Lecce, Italy; elisa.panzarini@unisalento.it (E.P.); bernardetta.tenuzzo@unisalento.it (B.A.T.); diana.vardanyan@unisalento.it (D.V.); 2 Consiglio Nazionale delle Ricerche (CNR) NANOTEC istituto di Nanotecnologia-Istituto di Nanotecnologia, 73100 Lecce, Italy; stefano.leporatti@nanotec.cnr.it (S.L.); alessandra.quarta@nanotec.cnr.it (A.Q.); 3 Nanomedicine Department, Institute of Nanoscience and Nanotechnology, Kafrelsheikh University, Kafr El Sheikh 6860404, Egypt; nemany.hanafy@nano.kfs.edu.eg; 4 National Institute of Gastroenterology S. De Bellis, IRCCS Research Hospital, Via Turi 27, 70013 Castellana Grotte, Italy; gianluigi.giannelli@irccsdebellis.it; 5 Department of Biology and Biotechnology “Charles Darwin”, Sapienza University of Rome, 00185 Rome, Italy; camilla.moliterni@uniroma1.it (C.M.); carolina.sbarigia@uniroma1.it (C.S.); 6 Research Center for Nanotechnology for Engineering of Sapienza (CNIS), Sapienza University of Rome, 00185 Rome, Italy

**Keywords:** hepatic fibrosis, cancer, LY2157299, galunisertib, TGF-β receptor, hepatic stellate cells

## Abstract

Hepatic fibrosis (HF) is a major cause of liver-related disorders and together with cancer-associated fibroblasts can favor liver cancer development by modulating the tumor microenvironment. Advanced HF, characterized by an excess of extracellular matrix (ECM), is mediated by TGF- β1, that activates hepatic stellate cells (HSCs) and fibroblasts. A TGF-β1 receptor inhibitor, LY2157299 or Galunisertib (GLY), has shown promising results against chronic liver progression in animal models, and we show that it can be further improved by enhancing GLYs bioavailability through encapsulation in polymeric polygalacturonic-polyacrylic acid nanomicelles (GLY-NMs). GLY-NMs reduced HF in an in vivo rat model of liver fibrosis induced by intraperitoneal injection of CCl_4_ as shown by the morphological, biochemical, and molecular biology parameters of normal and fibrotic livers. Moreover, GLY-NM was able to induce recovery from HF better than free GLY. Indeed, the encapsulated drug reduces collagen deposition, hepatic stellate cells (HSCs) activation, prevents fatty degeneration and restores the correct lobular architecture of the liver as well as normalizes the serum parameters and expression of the genes involved in the onset of HF. In summary, GLY-NM improved the pharmacological activity of the free TGF- β1 inhibitor in the in vivo HF treatment and thus is a candidate as a novel therapeutic strategy.

## 1. Introduction

Chronic liver disease (CLD) refers to a progressive deterioration of the liver characterized by a chronic inflammation that leads to hepatic fibrosis (HF), cirrhosis and, in the most critical condition, to liver cancer. Several factors are involved in the pathogenesis of the disease including both hepatitis B and C viral infections, and non-infectious factors such as alcohol abuse, metabolic disorders, steatosis, cholestasis, etc. CLD has a worldwide prevalence and the limited treatment options currently available make it a global health issue. 

HF is a dynamic process characterized by an altered deposition and an accumulation of extracellular-cell-matrix (ECM) (e.g., type I and III collagen, fibronectin, laminin) in the space of Dissè, between hepatocytes and sinusoids [1,2,3]. The liver architecture is distorted by the abundant ECM forming fibrous scars that, as a cascade, induces liver cell inflammation, hepatocyte damage and retention of inflammatory cells. Deposition of ECM alters the mechanical properties of the liver and modulates and coordinates multiple signalling networks in epithelial, endothelial, stromal, immune, and tumour cells by directly binding specific receptors, such as integrins and growth factor receptors [4]. HF and cancer-associated fibroblasts can modulate the tumour microenvironment and thus can influence liver cancer development [5,6].

An excess of structural ECM components in liver matrix is also associated with a structural change in the space of Dissè from collagens type V and VI to collagens type I and III and fibronectin. In fact, the activation of hepatic stellate cells (HSCs) during fibrosis induction replaces the physiologic matrix with fibrillar collagens [1,7]. This process elicits the obliteration of fenestrae in liver sinusoidal endothelial cells (LSECs) modifying them into a continuous vascular type, termed LSECs capillarization, leading to the formation of a basement membrane which is discontinuous in healthy conditions [8,9,10]. SECs capillarization interferes with the normal nutrient transport between sinusoidal blood and hepatocytes [7] whose exacerbation causes later stages of liver disease, liver cirrhosis and hepatocellular carcinoma (HCC), contributing to liver failure and death [11].

In theory, the removal of the causative factors of HF along with a specific anti-fibrotic therapy should be sufficient to manage CLD and prevent cancer onset [7,12]. Unfortunately, a successful approach is not yet currently available, mainly because of the difficulty inreaching and delivering therapeutic concentrations of drugs to the damaged liver [13,14]. Thus, there is an urgent need for both effective anti-fibrotic drugs and efficient delivery systems.

Among the mediators that act as pro-fibrotic inducers, TGF-β1 [15,16] is the most interesting for inducing both HSCs and portal fibroblasts to produce excessive ECM components. To understand HF it is necessary to understand the HSCs activation mechanism [17,18]. Indeed, HSCs, once activated, proliferate and transdifferentiate from retinoid storing cells to ECM-producing myofibroblasts (MFB), and can be the major source of liver ECM [19]. Therefore, inhibition of the pro-fibrotic effect of TGF-β1 is considered a promising therapeutic strategy against HF and the further progression of liver damage. Galunisertib (also known as LY2157299) (GLY), is a TGF-β receptor type I kinase inhibitor with antifibrotic properties. GLY is a very small molecule active against several types of cancers [20,21,22,23], which is currently being used in a phase II clinical trial carried out on HCC patients, in combination with Sorafenib (a multi-tyrosine kinase inhibitor) or with Nivolumab (a human IgG4 monoclonal antibody blocking PD-1) [22]. Alternative therapies for the inhibition of the TGF-β1 receptor, including chemical drugs, herbal medicines and monoclonal antibodies [12,24], aim to remove harmful stimuli, suppress liver inflammation, downregulate HSCs activation, and promote matrix degradation [25]. Unfortunately, these treatments exhibit limited therapeutic efficiency, and the side effects exclude routine use in clinical practice. 

Despite the promising properties of GLY (modulating the biochemical composition of the deposed ECM in a CLD mouse model (Abcb4ko)), some downsides, such as its lack of success at reducing the stage of HF, need to be addressed [26]. In fact, overcoming the drawbacks of therapeutic dosage at the target site because of blood dilution, retention by the immune system or loss in the gastrointestinal tract, especially for GLY that has inhibitor effects on signalling pathways, lies in the nanocarriers encapsulation [27]. Indeed, even if nanotechnology offers great opportunity, and many therapeutic or diagnostic engineered nanoparticles (NPs) agents for HF, such as metal and metal oxide NPs [28], lipid [29], polymer NPs [30], and protein NPs [31] have been developed [27,32,33], only a limited number of NPs are currently studied in preclinical trials and very few are already approved in clinical trials for HF [27,34]. It is interesting to note that the liver, due to its high vascularization and capacity to remove nutrients, metabolites, and particulate material from the bloodstream, shows a high passive accumulation of NPs. Indeed, hepatic sinusoidal fenestrations allow NPs up to a diameter of ~100 nm to be trapped in the space of Disse and thus, even without specific-targeted ligands, to be found in proximity to HSCs and thus target antifibrotic drugs directly to the main site of fibrogenesis [35]. In particular, hyaluronic acid micelles show liver targeting which is emphasized in the case of the HF [36]. In the treatment of HF and liver cancer much attention has been given to liposomes and NPs with stimulus-sensitive polymers, where cargo targeting and release can be triggered by specific internal or external stimuli [37,38,39,40].

Taking into account that a polymeric nanomicelle (polygalacturonic acid, PgA, and polyacrylic acid, PAA) formulation loaded with LY2157299 gave promising results in targeting and uptake in an HCC line, i.e., HLF [41], in the present work the possible in vivo use of GLY-NMs against CLD has been assayed. The study has focused on the stage of the onset of HF and the efficacy of GLY-NMs against HF, in a CCl_4_-induced HF rat model by comparing the data with the therapeutical properties of free GLY.

## 2. Materials and Methods

### 2.1. Chemicals

All the chemicals not specifically indicated were purchased from Sigma Aldrich (St Louis, MI, USA).

### 2.2. Animal Ethics and Procedures

The present study was approved by the Italian Ministry of Health (Ministero della Salute) (certificate no. 649/2016-PR, 1 July 2016). Experiments were carried out in accordance with local and national guidelines for animal experimentation.

Male Sprague-Dawley (SD) rats aged 8 weeks, weighing 150–200 g, were purchased from the Charles River Laboratories Italia, SRL (Lecco, Italia). All animals were housed under standard laboratory conditions in an animal holding room located at the Department of Biological Sciences and Technologies (Di.S.Te.B.A.), University of Salento (certificate no.48/2004-A). The acclimatization period was not less than one week after delivery. They were fed with standard chow diet and water was provided ad libitum. They were housed in small groups in plastic-bottomed cages containing sawdust. They were bred in a temperature-controlled environment (20 ± 2 °C) with 12 h of light/dark cycles (light from 6:00 a.m. to 6:00 p.m.). At specific time points, they were deeply anesthetized using isoflurane (drop jar method by placing an impermeable mesh grid over the cotton/gauze) and sacrificed through rapid dislocation of the cervical spine. 

Body weight, liver weight and Hepatic Index (HI) (percentage of liver weight with respect to body mass) were monitored.

### 2.3. Synthesis and Characterization of Polymeric Nanomicelles

Nanomicelles (NMs) synthesis, loading procedure, and characterization of GLY- loaded nanomicelles (GLY-NMs) were performed according to Hanafy and co-workers [41]. Briefly, 5 mL of polygalacturonic acid-R6G (PgA-R6G) and 4 mL of polyacrylic acid (PAA) were mixed for 30 min in constant rotation by using a magnetic stirrer and subsequently centrifuged at 13,000 rpm for 1 h. Micelles were collected and stored at +4 °C until use. 

### 2.4. Experimental Design

This study was designed to evaluate the therapeutic properties of GLY-NMs in liver fibrosis-induced rats following CCl_4_ injections (CCl_4_-induced HF rat model) [42,43]. Evaluation of the safety of the empty NMs was monitored. All animal groups were fed with standard chows.

**Figure 1:**
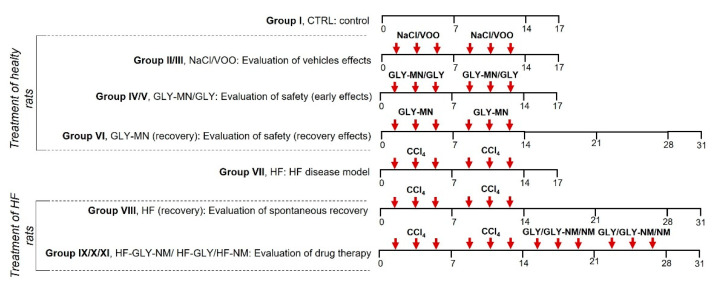
Schematic representation of the experimental groups. The horizontal axis indicates days of treatment and the last number on the horizontal axis is the day of the animal sacrifice. Arrows indicate intraperitoneal injections and the drug and/or solution administered (NaCl: sterile 0.9% NaCl; VOO: sterile virgin olive oil; GLY-MN: encapsulated LY2157299; GLY: free LY2157299; CCl_4_: CCl_4_, 40% in sterile virgin olive oil.

Rats were randomly assigned into 11 groups (n = 8 per group) as reported below. A schematic representation of the experimental groups is reported in Figure 1.

Group I, CTRL: Rats did not undergo treatment.

Group II, NaCl: Rats received intraperitoneal injections (500 µL/100 g body weight) of sterile 0.9% NaCl solution three times a week for 2 weeks. Animals were sacrificed 72 h after the last injection.

Group III, VOO: Rats received intraperitoneal injections (500 µL/100 g body weight) of sterile virgin olive oil three times a week for 2 weeks. Animals were sacrificed 72 h after the last injection.

Group IV, GLY-NM: Untreated rats administered with encapsulated Galunisertib-LY2157299 (GLY-NM) (GLY-NM safety assessment). Animals were intraperitoneally injected with (500 µL/100 g body weight) of encapsulated Galunisertib-LY2157299 (2.3 µmol/Kg) in sterile NaCl 0.9% solution three times a week for 2 weeks. Animals were sacrificed 72 h after the last injection.

Group V, GLY: Untreated rats administered with free Galunisertib-LY2157299 (GLY) alone (GLY safety assessment). Animals were intraperitoneally injected with (500 µL/100 g body weight) of free Galunisertib-LY2157299 (150 mg/kg in sterile NaCl 0.9% solution) in sterile NaCl 0.9% solution three times a week for 2 weeks. Animals were sacrificed 72 h after the last injection.

Group VI, GLY-NM (recovery): Untreated rats administered with encapsulated Galunisertib-LY2157299 (GLY-NM) (GLY-NM safety assessment). Animals were intraperitoneally injected with (500 µL/100 g body weight) of encapsulated Galunisertib-LY2157299 (2.3 µmol/kg) in sterile NaCl 0.9% solution three times a week for 2 weeks, followed by an additional 2 weeks with free access to food and water. Animals were sacrificed 17 days after the last injection.

Group VII, HF: Hepatic fibrosis (HF) model. Rats received intraperitoneal injections (500 µL/100 g body weight) of CCl_4_ (40% in sterile virgin olive oil) three times a week for 2 weeks. Animals were sacrificed 72 h after the last injection.

Group VIII, HF (recovery): Control of immune system response in an HF model. Rats received intraperitoneal injections (500 µL/100 g body weight) of CCl_4_ (40% in sterile virgin olive oil) three times a week for 2 weeks, followed by an additional 2 weeks with free access to food and water. Animals were sacrificed 17 days after the last injection.

Group IX, HF-GLY-NM: HF rats treated with encapsulated Galunisertib-LY2157299 (GLY-NM) (proposed therapeutic model). Animals, after intraperitoneal injections (500 µL/100 g body weight) of CCl_4_ (40% in sterile virgin olive oil) three times a week for two weeks were intraperitoneally injected with GLY-NM (2.3 µmol/kg) diluted in sterile 0.9% NaCl solution three times a week for two weeks. Animals were sacrificed 72 h after the last injection.

Group X, HF-GLY: HF rats treated with free Galunisertib-LY2157299 (GLY) (positive control of proposed therapeutic model). Animals, after intraperitoneal injections (500 µL/100 g body weight) of CCl_4_ (40% in sterile virgin olive oil) three times a week for 2 weeks were intraperitoneally injected with free GLY (150 mg/kg in sterile NaCl 0.9% solution) three times a week for 2 weeks. Animals were sacrificed 72 h after the last injection.

Group XI, HF-NM: HF rats treated with empty micelles (negative control of proposed therapeutic model). Animals, after intraperitoneal injections (500 µL/100 g body weight) of CCl_4_ (40% in sterile virgin olive oil) three times a week for 2 weeks were intraperitoneally injected with empty micelle (2.3 µmol/kg) in sterile NaCl 0.9% solution three times a week for 2 weeks. Animals were sacrificed 72 h after the last injection.

Because treatments were performed by intraperitoneal (IP) injection and CCl_4_, GLY and NMs were diluted in different solvents, we established some groups of controls to exclude possible animal sickness/adverse events due to the injection or solvents (Group I CTRL, untreated animals; Group II NaCl, IP injections of sterile 0.9% NaCl in untreated rats; Group III VOO, IP injections of sterile virgin olive oil in untreated rats).

The safety of GLY-NM and GLY was tested in untreated animals (Group IV GLY-NM, IP injections of encapsulated LY2157299 in untreated rats; Group V GLY-NM, IP injections of free LY2157299 in untreated rats). The possible late immune inflammation response due to GLY-NM was evaluated after treatment with the encapsulated drug following 2 weeks of recovery (Group VI GLY-NM (recovery), IP injections of encapsulated LY2157299 in untreated rats and recovered for 2 weeks). Liver fibrosis grade was evaluated both at the end of CCl_4_ treatment (Group VII HF, IP injections of CCl_4_) and after 2 weeks of recovery after the last injection of CCl_4_ (Group VIII HF (recovery), IP injections of CCl_4_ in untreated rats and recovered for 2 weeks). The efficacy of GLY treatment and the possible improvement due to its encapsulation in NM against liver fibrosis was determined by comparing rats administrated with CCl_4_ for 2 weeks and subsequently treated with GLY-NM (Group IX HF-GLY-NM, IP injections of encapsulated LY2157299 in CCl_4_ treated rats), GLY (Group X HF-GLY, IP injections of free LY2157299 in CCl_4_ treated rats) and NM (Group XI HF-NM; IP injections of empty micelles in CCl_4_ treated rats). 

### 2.5. Determination of Serum Biomarker for Liver Damage

At specific time points, blood samples collected from the heart of animals by heparinised needle, were centrifuged at 8000× *g* for 10 min, and subsequently, serum was collected from the supernatant.

Serum level of Alanine Transaminase (ALT), Aspartate Transaminase (AST) and Lactate Dehydrogenase (LDH) were measured using commercial kits available according to manufacturers’ instruction (ALT: Cayman Chemical, Ann Arbor, MI, USA; AST and LDH: Sigma Aldrich, St Louis, MO, USA).

### 2.6. Histological Preparation and Examination

Liver specimens were fixed in 4% formalin in phosphate buffer saline (PBS), dehydrated with crescent graded alcohol (30, 50, 70, 90 and 100% *v/v*), incubated in xylene and then embedded in paraffin at 63 °C. Sections of 5 µm thickness were obtained using a microtome. Liver slices were deparaffinized, rehydrated and stained with haematoxylin and eosin (H & E) for morphological analysis and histopathological changes [44]. Liver slices stained with Masson’s trichrome staining were observed for detecting collagen deposition (interstitial fibrosis). Quantification of interstitial fibrosis was performed by measuring collagenous fibrotic areas stained in blue/green by Masson’s trichrome staining in 10 random liver fields/section from images taken at a magnification of 20× using multiphase image analysis with ImageJ software version 1.49 s [45].

For immunofluorescence, samples of the liver were fixed by immersion in PFA (4% in PBS) for 2 h and then dehydrated and embedded in 54 °C paraffin. Sections of 5 mm thickness were cut by using a microtome Reichert-Jung 2050 Supercut (Leica Microsystems GmbH, Wetzlar, Germany). Immunohistochemical analysis of alpha-SMA was performed on the liver sections placed onto gelatine-coated slides. Slides were incubated with blocking solution (3% BSA in PBS) for 30 min at room temperature in a humidified chamber to block excess proteins and prevent nonspecific antibody binding, and then incubated with trypsin 1X in PBS for 5 min at 37 °C for antigen retrieval. After permeabilization with 0.2% Triton-X100/PBS for 6 min at 4 °C, sections were incubated overnight at 4 °C with alpha-SMA primary polyclonal antibody (1:100, SAB1400414, Sigma-Aldrich, St Louis, MI, USA), washed three times with PBS and then incubated with FITC conjugated secondary antibody (1:50, F6257 Sigma-Aldrich, St Louis, MI, USA) in the dark for 1 h at room temperature. After extensive washing with PBS, liver sections were mounted with fluorescent mounting media (F4680, Fluoromount™ Aqueous Mounting Medium, Sigma-Aldrich, St Louis, MI, USA). The specificity of immunostaining was tested by incubation without the primary or/and secondary antibodies.

### 2.7. RNA Preparation and RT-qPCR Analysis

RNA was extracted from formalin-fixed liver paraffin-embedded specimens modifying Choi’s and co-workers’ protocol [46]. Briefly, paraffin blocks were cut (approximately 10 µm), deparaffined in xylene and washed in alcohol 100%. After alcohol evaporation, tissue was treated with protease K for 3 h at 55 °C in 150 mL protease K buffer (500 μg/mL protease K, 20 mM Tris-HCl pH 8.0, 1 mM CaCl_2_, 0.5% sodium dodecyl sulfate hydrated). Samples were subsequently used for RNA extraction using TRIzol reagent (Invitrogen) according to the manufacturer’s instructions. Following this, mRNA was quantified at NanoDrop (ThermoFisher, Waltham, MA USA). Reverse transcription was performed by Bioline SensiFAST cDNA Synthesis Kit (Aurogene, Rome, Italy) according to the user’s manual using 1 µg of RNA for each sample. The amount of 50 ng of cDNA-equivalent RNA was used for RT-qPCR. RT-qPCR amplification was performed using Bioline SensiFAST SYBR Lo-ROX Kit (Aurogene, Rome, Italy). Primers are listed in Table S1.

### 2.8. Protein Extraction and Western Blotting Analysis

Protein extraction from formalin-fixed paraffin-embedded tissues was performed according to Guo and co-workers [47]. Briefly, paraffin blocks were cut (approximately 10 µm), deparaffined in xylene and rehydrated in decrescent graded alcohol (100, 90, 70, 50, 30% *v/v*). Samples were resuspended in 20 mM Tris pH 9 and 2% SDS and boiled for 20 min at 100 °C and 2 h at 80 °C. After centrifugation, supernatants were collected, and protein content quantified by Quick Start Bradford (Bio-Rad, Hercules, CA, USA). Fifty μg of proteins from each sample were diluted in Laemmli buffer and boiled for 5 min. Samples were subsequently resolved on SDS-PAGE and transferred to PVDF membranes. Membranes were blocked for 1 h at RT with 5% non-fat dry milk in TBS buffer (20 mM Tris and 105 mM NaCl) containing 0.1% Tween-20 (block solution) and then incubated overnight with primary antibody dissolved in block solution. The day after, membranes were washed three times in TBS containing 0.1% Tween-20 (TBST) and incubated in secondary anti-mouse IgG conjugated to horseradish peroxidase (Bio-Rad, Hercules, CA, USA) diluted 1:10,000 in block solution. Immunostained bands were detected by a chemiluminescent method (Clarity Western ECL Substrate, #1705061, Bio--ad, Hercules, CA, USA). Primary antibodies (α-SMA and tubulin) were diluted 1:1000 in block solution.

### 2.9. Image Acquisition

Images were acquired using a Nikon 80i microscope (Nikon Eclipse Nikon, Tokyo, Japan) equipped with a digital camera Nikon DXM1200F (Nikon Eclipse Nikon, Tokyo, Japan). 

### 2.10. Statistical Analysis

Multiple comparisons were performed by two-way ANOVA. Comparisons between two groups were performed using a Student’s *t*-test (GraphPad Prism 7 software, GraphPad Software, San Diego, CA, USA). Eight animals for each experimental group were used. Data are presented as a mean value ± SD and all tests were performed at the 0.05 significance level.

## 3. Results

### 3.1. Body and Liver Weight and Hepatic Index 

Empty and LY2157299 (GLY) loaded polymeric nanomicelles were round in shape, not clustered and drug encapsulation efficiency was 23% in accordance with previous work [41]. Body weight (BW), liver weight (LW) and Hepatic Index (HI) monitored for all experimental groups are reported in Table 1. Animals injected with only the vehicle of drugs, i.e., sterile virgin olive oil (Group III VOO) as well as sterile 0.9% NaCl (Group II NaCl), showed no alteration of BW, LW and HI when compared with control animals (Group I CTRL). In addition, free GLY and GLY-NM were ineffective at modulating BW, LW and HI when administered to healthy rats (Group IV GLY-NM, Group V GLY) and two weeks after the end of GLY-MN treatment (Group VI GLY-MN late). Indeed, alterations of BW, LW or HI were observed in the group of rats induced to HF by intraperitoneal injections with CCl_4_ (Group VII HF). In particular, Group VII HF showed reduced HI and LW but not BW. Two weeks after the last injection of CCl_4_ (Group VIII HF recovery), a further dramatically reduction of BW and LW but not of HI, still comparable to Group VII HF, was observed. The administration of the empty micelles after CCl_4_-induced HF (Group XI HF-NM), did not recover normal values of BW, LW and HI. Thus, these alterations are entirely attributable to CCl_4_ treatment and suggest that empty micelles had no therapeutic or adverse proprieties in our disease model. Both free GLY and GLY-NM showed therapeutic effects when administrated to HF rats by normalizing the HI value to control rats (Group IX HF GLY-NM and Group X HF-GLY). Interestingly, only animals treated with GLY-NM but not with free GLY after CCl_4_-induced HF showed a BW similar to control rats, indicating a better therapeutic action of the encapsulated drug in the recovery of healthy conditions. 

Based on these results further analyses were focused on Group I CTRL, Group VII HF, Group IX HF GLY-NM and Group X HF GLY. 

### 3.2. GLY-NM Normalizes Liver Morphology and Function after CCl_4_-Induced HF

**Figure 2:**
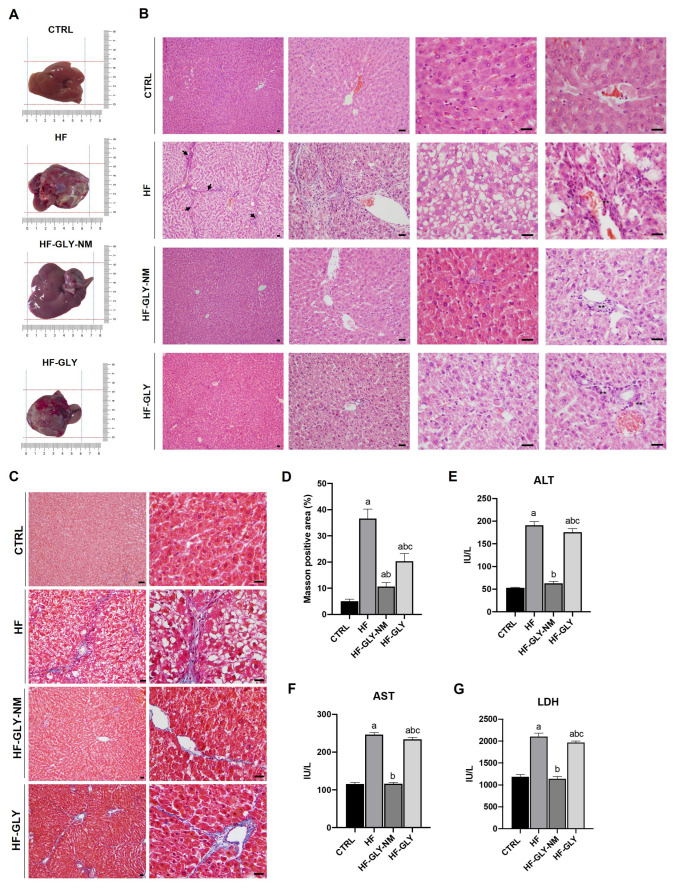
Effects of GLY and GLY-MNs on liver morphology and function. (**A**) Representative images of livers from CTRL (Group I), HF (Group VII), HF-GLY-NM (Group IX) and HF-GLY (Group X). (**B**) Haematoxylin and eosin staining of liver sections. Arrow indicates typical irregular shape of liver lobules during fibrosis (scale bar: 10 μm). (**C**) Masson’s trichrome staining of liver sections (scale bar: 10 μm). (**D**) Quantification of Masson’s trichrome positive areas. The amount of interstitial fibrosis was measured by quantifying the collagenous fibrotic areas stained in blue/green in 10 random liver fields per section from four different rats for each group. Images were analysed by using multiphase image analysis ImageJ with a software version 1.49s. Values are expressed as mean ± SD. (**E**) Alanine Transaminase (ALT), (**F**) aspartate transaminase (AST) and (**G**) lactate dehydrogenase (LDH) activities. Values are expressed as mean ± SD (n = 8). Significative values were reported with respect to Group I CTRL, a = *p* < 0.05; to Group VII HF, b = *p* < 0.05; to Group IX HF-GLY-NM, c = *p* < 0.05.

Macroscopic morphological comparison of livers (Figure 2A) highlighted that livers from fibrotic rats (HF, Group VII) were pale, hard, rough, and shrunken thus indicating serious damage. Administration of GLY to Group VII animals slightly mitigated the morphological alteration while treatment with GLY-MN re-established normal colour, size, and texture of the liver (Figure 2A).

H & E and Masson’s histological analysis of livers from groups I, (CTRL, rats did not undergo treatment), II (NaCl, rats received intraperitoneal injections of sterile 0.9% NaCl) and III (VOO, rats received intraperitoneal injections of sterile virgin olive oil) did not show alteration in the typical hexagonal shape of lobule or in the extracellular matrix distribution (Supplementary Figure S1A). On the contrary, livers from CCl_4_-induced HF showed features of tissue injury in the hepatocytes as necrosis, macrosteatosis, ballooning, transparent cytoplasm, and nuclei localized at the cell periphery (Figure 2B and Table 2). Increase in apoptosis in comparison to normal livers was not morphologically observed. Wide or narrow stripes of collagen fibres in the extracellular matrix divide the liver parenchyma into lobules of unequal size and irregular shape, i.e., pseudolobules. In addition, substantial inflammatory cells infiltration in the extracellular matrix, which is considered one of the main HF features, was observed only in animals of Group VII-HF (Figure 2B). The abnormal deposition of collagen fibres was highlighted by Masson’s trichrome staining in HF rat liver and the image quantification of the Masson’s staining positive area revealed a dramatic increase with respect to control animals (Group I CTRL) (Figure 2C,D). Altogether, abundant extracellular matrix and the presence of pseudolobules, confirm the onset of CCl_4_-induced HF in our animal model. 

Administration of GLY in fibrotic rats (Group X HF-GLY) reduced balloon-like degeneration, necrosis, and inflammatory cell infiltrations in the portal area, pseudolobules with fibrous septa (Figure 2B); on the contrary, although a slight reduction of collagen fibres surrounded the hepatic vein, these were still present at a relevant amount (Figure 2C,D). Conversely, after treatment with GLY-NM (Group IX HF-GLY-NM) a substantial reduction of liver damage on the histological architecture caused by CCl_4_ treatment was observed (Figure 2B–D). In particular, the collagen amount decreased to levels comparable with untreated rats, and only a scant presence of inflammatory cells was still present. The administration of empty MNs to HF rats or two weeks of recovery after the last CCl_4_ injection was not effective in restoring liver morphology, thus excluding that improvement observed after drug treatment was not dependent on the nanocarrier or on self-healing (Supplementary Figure S1A,B). Furthermore, comparing the livers of CCl_4_-induced HF animals (Group VII) subjected to drug treatment, i.e., with GLY or with GLY-MN (Group X and Group IX) or rats after two weeks of recovery (Group VIII HF, recovery), the appearance of balloon-like degeneration of hepatocytes is hampered by drug administration (GLY-MN more than GLY) (Figure 2B; and Supplementary Figure S1A) and macroscopic analysis of livers suggest a reduction in fat accumulation after GLY-NM treatment (Figure 2B,C). In addition, by comparing the liver architecture of Group I CTRL with those of drug-treated animals, it is evident how the lobule structure is recovered with a higher efficacy with GLY-NM compared to GLY (Figure 2B–D). 

Accordingly, the serum levels of ALT, AST and LDH were significantly increased in CCl_4_-induced HF (Figure 2E–G) and the serum levels of ALT, AST and LDH were normalized to the values of control rats when fibrotic animals were treated with GLY-MN while free GLY was not effective. 

### 3.3. Drug Effects on HSCs Activation, Protein and Gene Liver Expression in CCl_4_-Induced HF 

**Figure 3:**
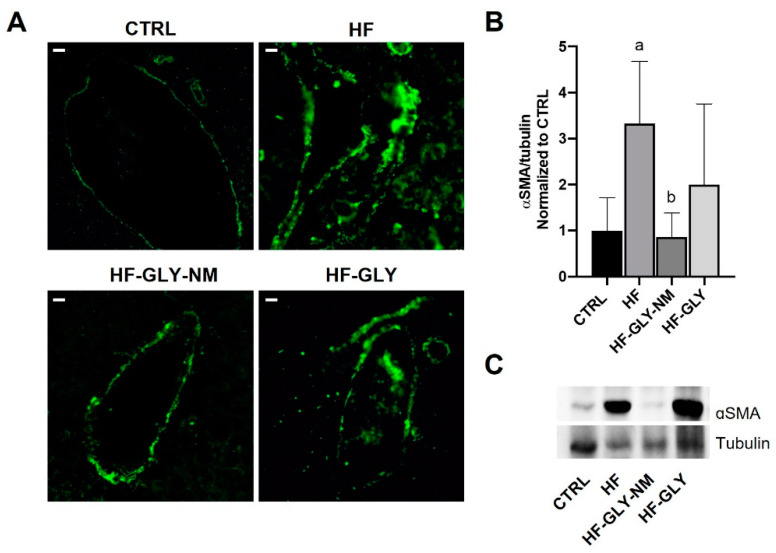
Immunolocalization of α-smooth muscle actin expression (α-SMA). (**A**) Fluorescent pictures of immunostaining of α-SMA in liver sections from CTRL (Group I), HF (Group VII), HF-GLY-NM (Group IX) and HF-GLY (Group X). Scale bars = 10 μm. (**B**) Western blot of α-SMA. Values are expressed as a fold change with respect to CTRL ± SD (n = 3). Tubulin was used as the loading control and for band density normalization. Significative values were reported with respect to Group I CTRL, a = *p* < 0.05; to Group VII HF, b = *p* < 0.05. (**C**) Representative western blot of α-SMA. (Group I): Control untreated rats allowed free access to food and water; HF (Group VII): rats intraperitoneal injected with CCl_4_ three times a week for two weeks; HF-GLY-NM (Group IX): HF rats treated with encapsulated Galunisertib-LY2157299 three times a week for two weeks following CCl_4_ injections; HF-GLY (Group X): HF rats treated with free Galunisertib-LY2157299 three times a week for two weeks following CCl_4_ injections.

Activation of HSCs is another feature of liver fibrosis that was checked by immunofluorescence of α-SMA [48]. Livers from control animals (Group I CTRL) showed a discrete positive α-SMA cells in the perisinusoidal spaces and vascular walls whose fluorescence intensity and area increased in the fibrotic liver as sign of HSCs activation, in agreement with Zhang and co-workers [48] (Figure 3A). In agreement with what they reported for liver morphology and function, the injection of GLY-NM, but not of GLY, to CCl_4_-induced HF rats significantly reduced α-SMA-positive area, thus suggesting a reduction of activated HSCs.

**Figure 4:**
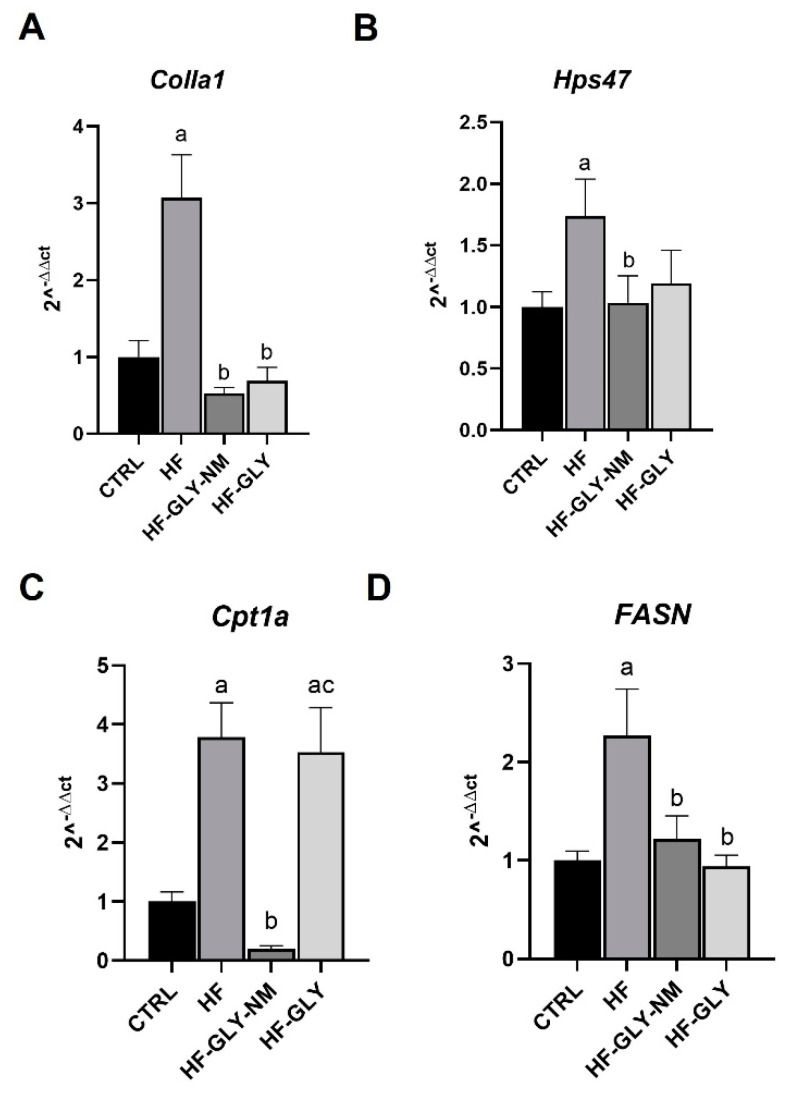
Gene expression of collagen type I, α 1 (*ColIa1*), heat shock protein 47 (*Hsp47*), fatty acid synthase (*FASN*) and carnitine palmitoyltransferase 1a (*Cpt1a*). Relative mRNA level of (**A**) *ColIa1*, (**B**) *Hsp47*, (**C**) *FASN* and (**D**) *Cpt1a* in liver from CTRL (Group I), HF (Hepatic fibrosis; Group VII), HF-GLY-NM (Group IX) and HF-GLY (Group X). Values are expressed as fold change with respect to CTRL ± SD (n = 3). *GADPH* expression levels were used as the housekeeping gene. (Group I): Control untreated rats allowed free access to food and water; HF (Group VII): rats intraperitoneal injected with CCl_4_ three times a week for two weeks; HF-GLY-NM (Group IX): HF rats treated with encapsulated Galunisertib-LY2157299 three times a week for two weeks following CCl_4_ injections; HF-GLY (Group X): HF rats treated with free Galunisertib-LY2157299 three times a week for two weeks following CCl_4_ injections. Significative values were reported with respect to Group I CTRL, a = *p* < 0.05; to Group VII HF, b = *p* < 0.05; to Group IX Hf-GLY-NM, c = *p* < 0.05.

To corroborate the histopathological analysis with quantitative data, the protein content of α-SMA and the expression of genes involved in HF, i.e., collagen type I, α 1(*ColIa1)* and heat shock protein 47 (*Hsp47)* were evaluated together with genes encoding for enzymes involved in fatty acid metabolism, i.e., fatty acid synthase (*FASN*) and carnitine palmitoyltransferase 1a (*Cpt1a*). *ColIa1*, *Hsp47* and α-SMA in the rat experimental models are induced by TGF-β1. TGF-β1 is a key profibrogenic cytokine in liver fibrosis which triggers the morphological hepatic changes and ECM production [49]. Biochemical data are in accordance with histopathological analysis. In fact, they are also in agreement with the increased fluorescence area of α-SMA in livers of CCl_4_-induced HF rats and also the 3-fold α-SMA protein increase, suggesting activation of HSCs (Figure 3A–C). After administration of the drug to CCl_4_-induced HF animals, the protein level of α-SMA was comparable to that observed in control rats when injected with GLY-NM. On the contrary, injection of GLY was not able to counteract the effects of CCl_4_ (Figure 3B,C). Moreover, only a partial recovery in the α-SMA protein values was observed similar to control rats (about 2-fold induction) in those rats left to recover for two weeks after the last CCl_4_ injection or treated with empty MN (Supplementary Figure S1C,D). *ColIa1* and *Hsp47* mRNA levels were higher in CCl_4_-induced HF animals than in control ones, confirming the fibrosis process (Figure 4A,B). When these animals were administrated with GLY or GLY-NM, both treatments reduced *ColIa1* and *Hsp47* to the same extent, thus not highlighting a difference, at least in the mRNA contents (Figure 4A,B).

The expression levels of two genes involved in fatty acid metabolism, i.e., *FASN* which participates in the synthesis of fatty acid and *Cpt1a* which participates in fatty acid oxidation were assayed (Figure 4C,D). The mRNA level of both *Cpt1a* and *FASN* were up-regulated in CCl_4_-induced HF indicating an imbalance in lipid homeostasis (Figure 4C,D). *FASN* mRNA of CCl_4_-induced HF rats was downregulated to levels comparable to control untreated animals by both GLY and GLY-MN treatment. Surprisingly, the mRNA level of *Cpt1a* was strongly reduced only after GLY-MN but not after GLY treatment. 

## 4. Discussion

In the present study, the antifibrotic properties of the TGF-β1 inhibitor LY2157299 (GLY), encapsulated in polymeric nanomicelles, were investigated in a rat model of CCl_4_-induced HF and compared with the effects of the administration of a free drug in the same HF animal model. 

The best strategy currently under investigation to reduce HF is represented by the inhibition of TGF-β1 receptor. The TGF-β1 receptor has a crucial role in the development of the disease because it is involved in the activation of HSCs that are the major source of ECM in liver fibrosis and cirrhosis [17]. In addition, the TGF- β1 pathway impacts on lipid homeostasis in liver fibrosis [50,51,52]. However, the efficacy of GLY on the TGF-β1 pathway can be hindered by several factors which hamper the efficient delivery of drug due to blood dilution, immune system cleansing and loss in the gastrointestinal tract [26,27].

Encapsulated GLY combines the advantages of nanocarrier-mediated drug delivery with the known beneficial effect against HF. The nanomicelles proposed in the present study are polymeric nanomicelles of PgA-PAA that encapsulate LY2157299 (GLY) at high efficiency with a satisfactory release rate [41]. Among the large number of nanocarriers currently under investigation, only a few are currently employed in clinical trials for liver fibrosis [27]. In this context, GLY-NM seems to be a promising candidate, considering the results presented here, in addition to those previously published [41].

The efficacy of the nanocarrier chosen in this study was tested in an in vivo rat model of liver fibrosis. HF was obtained by intraperitoneal injection of CCl_4_. Indeed, induction of HF by CCl_4_ still represents the most common method that can mimic human HF [53]. It gives a high reproducibility of results and is easily manageable (only ≤3 intraperitoneal injections of CCl_4_ for rats). 

CCl_4_ treatment leads to a combination of effects causing hepatocyte damage via lipid peroxidation, HSCs activation, Kupffer cells activation, TGF-β1 upregulation and increased oxidative stress [54]. The achievement of the HF in our experimental conditions was validated by macroscopic and microscopic morphological analyses, biochemical evaluations, and alterations in the expression of the genes involved in the HF processes. The observed changes are essentially attributable to morphological changes such as characteristic pseudolobules, steatosis with a necrotic area, intense inflammation, and formation of thick fibrous septa due to high collagen fibre deposition [55]. In addition, the biochemical and molecular results regarding ALT, AST and LDH levels and *ColIa1*, *Hsp47, Cpt1a* and *FASN* mRNA expression have confirmed the fibrotic status in animals treated with CCl_4_ and are in line with other work in the literature [1,2,3]. Again, as reported by Fortea and co-workers [56] the CCl_4-_ induced HF was further confirmed by the reduction of BW, LW and HI. Finally, activation of HSCs, another feature of the HF, was detected in our model of HF as abundant α-SMA-positive areas. Even if the experimental procedure involving in vitro isolated HSCs, i.e., primary as well as cell line, represent a major advance in exploring the complex mechanisms involved in fibrogenesis, only animal models permit the study of the fibrosis occurrence and treatment on the basis of real interactions occurring between different cell types in the liver [53]. In disagreement with the report of Rooshi and colleagues [3] in which the formation of fibrosis regresses when CCl_4_ treatment is stopped, our data show that after two weeks of recovery the liver still presents with characteristic signs of fibrosis. 

Even if the nature of polymeric micelles does not allow their direct identification within the liver, the internalization of the encapsulated drug can be indirectly deduced using the positive recovery responses from signs of HF. The responses of the fibrotic animals treated with the free or encapsulated drug are different (encapsulated GLY is better than free GLY), while the empty carrier does not induce any alteration of the analysed parameters. The remarkably positive effect of GLY encapsulated in the polymeric micelles could be ascribed to the chemical properties of the polymers. Indeed, PgA is a natural polymer with limited solubility in water and it is not degradable in the upper gastrointestinal tract [57]. The strategy of combining PgA with PAA, a hydrophilic polymer [58], confers swelling properties to the complex and allows a great loading efficiency (about 23%) of GLY. Furthermore, the nano-elastic properties of PgA–PAA micelles allow their use not only for oral but also for systemic administration [41]. Probably due to their ability to enter activated HSCs directly through the fenestrae and the intrinsic ability of Kupffer cells to internalize circulating particles, GLY-MNs are more effective than non-encapsulated drugs against CCl_4_-induced HF. Liver from fibrotic rats treated with GLY showed a normal HI value, a reduction of balloon-like degeneration, necrosis, inflammatory cells in the portal area and pseudolobules with thick fibrous septa. On the contrary HSCs activation continued, thus favoring ECM deposition; BW and LW were high. The amount of α-SMA proteins decreased after GLY administration but values were still higher than the control rats. These results are in line with those of other preclinical animal studies on liver fibrosis using GLY [26,49,52]. If GLY showed beneficial effects against HF, those observed after treatment with GLY-MN were much stronger in ameliorating the damages of CCl_4_-induced HF. Indeed, after treatment with GLY-MN of CCl_4_-induced HF, macroscopic liver morphology, ALT, AST and LDH blood values, BW, LW and HI recovered to normal condition. Accordingly, pseudolobules, ballooning hepatocytes, fatty imbalance, abnormal accumulation of collagen, and activated HSCs dramatically decreased. Of note is the trend to normality of α-SMA protein and *ColIa1* and *Hsp47* mRNA levels. Although only GLY-MN was able to restore α-SMA content to values comparable to control rats, *ColIa1* and *Hsp47* mRNA were reduced to the same extent by both treatments. This can be ascribed to the different threshold drug doses needed to induce change in the considered markers. Luangmonkong and co-workers [49], in an ex-vivo precision-cut liver slice model performed a dose-response assay using GLY, showed that *ColIa1* and *Hsp47* mRNA downregulation is caused by a lower concentration of GLY than the one needed to decrease α-SMA level. Thus, *ColIa1* and *Hsp47* gene expression is inhibited by a low dose of GLY, while α-SMA is inhibited by a high one. Therefore, in the light of the data of Luangmonkong and colleagues [49], the trend of *ColIa1* and *Hsp47* mRNA and α-SMA in our rat model suggests that a greater effect of GLY on the fibrotic liver is obtained when the drug is encapsulated. Indeed, nanomicelles increased the drug delivery at the damaged liver sites that is hampered by the complex three-dimensional structure of the fibrotic liver. Furthermore, liver diseases and CCl_4_-induced HF are characterized by a dysregulation of lipid metabolism [59] that was largely reduced by GLY-MN treatment. Data from the literature report that the expression of *Cpt1a* mRNA is up-regulated in CCl_4_-induced HF in mice and promote oxidative stress, while the silencing of *Cpt1a* (*Cpt1a^−/−^* mouse) protects against steatosis in CCl_4_-treated animals [60]. In agreement with this literature data [51,59,61] reported up-regulation of *FASN* genes in several hepatic fibrosis animal models, and our results indicate an up-regulation of *Cpt1a* and *FASN* genes. Both GLY and GLY-MN treatment restored *FASN* to levels comparable with untreated animals while, on the contrary, *Cpt1a* is strongly reduced only after GLY-MN, suggesting that the encapsulated drug contributed to a greater reduction in this gene, in line with the protective effect observed in the *Cpt1*^−/−^ mouse produced by Luo and co-workers [60]. GLY is a TGF-β receptor type I kinase inhibitor. The impact of the TGF-β1 pathway on lipid homeostasis has emerged in the last decade, as well as the impact of lipid homeostasis on liver fibrosis. Although several recent studies have investigated the possible role of TGF-β1 pathway on fat metabolism [50,52], further investigations are needed to delineate the fine mechanism that links GLY to a possible role in lipid homeostasis.

## 5. Conclusions

In conclusion, in the present study, data showing that administration of an inhibitor of TGF β1 attenuates damage caused by fibrosis in liver from rats treated with CCl_4_ have been reported. Blocking HF is a major step in avoiding cancerous transformation and nanoengineered TGFb1 inhibitors could play a crucial role in determining successful treatment. Altogether our results confirm the anti-fibrotic activity of the GLY, further emphasized by its encapsulation in polymeric nanomicelles. Hence, this study highlights the benefits of tailored lipid-based nanoformulations to improve drug efficacy, tissue targeting and specificity, representing a powerful new therapeutic tool for liver. Our results show a great potential for GLY-MN to reduce HF, a risk factor for cancer development. Further studies on hepatic cancer animal models could be useful to evaluate the role of GLY-MN in improving the hepatic microenvironment and reducing the progress of tumors in the late-stage of the disease. Indeed, GLY is currently in phase II evaluation for hepatocellular carcinoma treatment, thus the double property of GLY to be active against liver cancer and HF could be exploited during the reversible phases of liver disease and block or at least slow down the tumour progression. Moreover, future development could be envisaged in the area of surface functionalization, e.g., by conjugating\using specific molecules\polymers, providing an enhancement as a targeted delivery system or by specific labelling for cancer bio imaging, for example by conferring theranostics properties through synergically adding therapeutic and diagnostic moieties.

## Figures and Tables

**Table 1:** Body and liver weights and hepatic index. Values are expressed as mean ± SD (n = 8). Hepatic Index is expressed as the percentage of the liver weight with respect to the total animal weight. CTRL (Group I): Control untreated rats were allowed free access to food and water. NaCl (Group II): Rats intraperitoneal injected with sterile 0.9% NaCl solution three times a week for two weeks. VOO (Group III): Rats intraperitoneal injected with sterile virgin oil three times a week for two weeks. GLY-NM (Group IV): Untreated rats administered with encapsulated Galunisertib-LY2157299 (GLY-NMs) (GLY-NMs safety assessment); GLY (Group V), GLY: Untreated rats administered with free Galunisertib-LY2157299 (GLY) alone (GLY safety assessment); GLY-MN (recovery) (Group VI): Untreated rats administered with encapsulated Galunisertib-LY2157299 (GLY-NMs) followed by additional 2 weeks with free access to food and water (GLY-NMs safety assessment); HF (Group VII): Hepatic fibrosis (HF) model; HF (recovery) (Group VIII): HF rats were left to recover for two weeks after the last injection of CCl_4_ with free access to food and water (control of immune system responses); HF-GLY-NM (Group IX): HF rats treated with encapsulated Galunisertib-LY2157299 (GLY-NM) (proposed therapeutic model); HF-GLY (Group X): HF rats treated with free Galunisertib-LY2157299 (GLY) (positive control of proposed therapeutic model); HF-NM (Group XI): HF rats were intraperitoneal injected with empty micelles three times a week for two weeks after the last injection of CCl_4_ (negative control of proposed therapeutic model). Significative values were reported with respect to Group I CTRL, ^a^ = *p* < 0.05; to Group VII HF, ^b^ = *p* < 0.05; to Group IX HF-GLY-NM, ^c^ = *p* < 0.05.

Sample	Treatment	Body Weight (g)	Liver Weight (g)	HI (%)
Group I, CTRL	No treatment	312.54 ± 2.05	7.69 ± 2.42	2.46 ± 0.77
Group II, NaCl	IP injections of sterile 0,9% NaCl in untreated rats	313.69 ± 9.05	7.73 ± 0.42	2.46 ± 0.13 ^b^
Group III, VOO	IP injections of sterile virgin olive in untreated rats	308.46 ± 19.38	7.71 ± 0.25	2.49 ± 0.08 ^b^
Group IV, GLY-NM	IP injections of encapsulated LY2157299 in untreated rats	319.4 ± 11.03	7.71 ± 0.25	2.41 ± 0.08 ^b^
Group V, GLY	IP injections of free LY2157299 in untreated rats	315.23 ± 8.05	7.72 ± 1.25	2.44 ± 0.39 ^b^
Group VI, GLY-NM (recovery)	IP injections of encapsulated LY2157299 in untreated rats and recovered for 2 weeks	309.37 ± 24.32	7.79 ± 0.39	2.51 ± 0.12 ^b^
Group VII, HF	IP injections of CCl_4_	304.64 ± 8.45	4.69 ± 0.36 ^a^	1.53 ± 0.11 ^a^
Group VIII, HF (recovery)	IP injections of CCl_4_ in untreated rats and recovered for 2 weeks	218.48 ± 9.2 ^ab^	3.98 ± 0.19 ^a^	1,82 ± 0,09 ^a^
Group IX, HF-GLY-NM	IP injections of encapsulated LY2157299 in CCl_4_ treated rats	303.66 ± 4.65	7.29 ± 0.56 ^b^	2.4 ± 0.18 ^b^
Group X, HF-GLY	IP injections of free LY2157299 in CCl_4_ treated rats	251.97 ± 11.25 ^abc^	6.21 ± 0.51 ^abc^	2.46 ± 0.20 ^b^
Group XI, HF-NM	IP injections of empty micelles in CCl_4_ treated rats	215.24 ± 8.65 ^ab^	4.01 ± 0.29 ^a^	1.86 ± 0.13 ^a^

**Table 2:** Histopathological observations of livers. CTRL (Group I): Control untreated rats allowed free access to food and water. NaCl (Group II): Rats intraperitoneal injected with sterile 0.9% NaCl solution three times a week for two weeks. VOO (Group III): Rats intraperitoneal injected with sterile virgin oil three times a week for two weeks; GLY-NM (Group IV): Untreated rats administered with encapsulated Galunisertib-LY2157299 (GLY-NMs) (GLY-NMs safety assessment); GLY (Group V), GLY: Untreated rats administered with free Galunisertib-LY2157299 (GLY) alone (GLY safety assessment); GLY-MN (recovery) (Group VI): Untreated rats administered with encapsulated Galunisertib-LY2157299 (GLY-NMs) followed by additional 2 weeks with free access to food and water (GLY-NMs safety assessment); HF (Group VII): Hepatic fibrosis (HF) model; HF (recovery) (Group VIII): HF rats were left to recover for two weeks after the last injection of CCl_4_ with free access to food and water (control of immune system responses); HF-GLY-NM (Group IX): HF rats treated with encapsulated Galunisertib-LY2157299 (GLY-NM) (proposed therapeutic model); HF-GLY (Group X): HF rats treated with free Galunisertib-LY2157299 (GLY) (positive control of proposed therapeutic model); HF-NM (Group XI): HF rats were intraperitoneal injected with empty micelles three times a week for two weeks after the last injection of CCl_4_ (negative control of proposed therapeutic model).

Sample	Morphological Modifications
Group I, CTRL	None
Group II, NaCl	None
Group III, VOO	None
Group IV, GLY-NM	Presence of inflammatory cells infiltration
Group V, GLY	None
Group VI, GLY-NM (recovery)	Presence of inflammatory cells infiltration
Group VII, HF	Fibrosis after 2 weeks
	Increase of collagen fibers
	Presence of pseudolobuli
	High macrosteatosis
	High hepatocellular ballooning
	Increase of inflammatory cells infiltration
Group VIII, HF (recovery)	Fibrosis after 2 weeks
	Increase of collagen fibers
	Presence of pseudolobuli
	High macrosteatosis
	High hepatocellular ballooning
	Increase of inflammatory cells infiltration
Group IX, HF-GLY-NM	No fibrotic septa
	Presence of inflammatory cells
Group X, HF-GLY	No fibrotic septa
	High amount of collagen fibers around blood vessels
Group XI, HF-NM	Fibrosis after 2 weeks
	Increase of collagen fibers
	Presence of pseudolobuli
	High macrosteatosis
	High hepatocellular ballooning
	Increase of inflammatory cells infiltration

## Data Availability

The data that support the findings of this study are available from the corresponding authors, M.F., S.T. and L.D., upon reasonable request.

## Supplementary Materials

The following supporting information can be downloaded at: https://www.mdpi.com/2075-4426/12/11/1812/s1, Figure S1: Hematoxylin and eosin and Masson’s stainings. Liver sections from untreated rats; and treated with vectors; and CCl_4_-induced HF and treated with MNs or recovering.; Table S1: Primers used for RT-qPCR reaction.
