# Supplementary material for: Therapeutic Effect of Polymeric Nanomicelles Formulation of LY2157299-Galunisertib on CCl4-Induced Liver Fibrosis in Rats
Source: J Pers Med. 2022 Nov 1;12(11):1812. doi: 10.3390/jpm12111812 (PMC9692463; doi:10.3390/jpm12111812)
Supplement: Supplementary file 1 [file jpm-12-01812-s001.zip › jpm-1945215-supplementary.pdf]

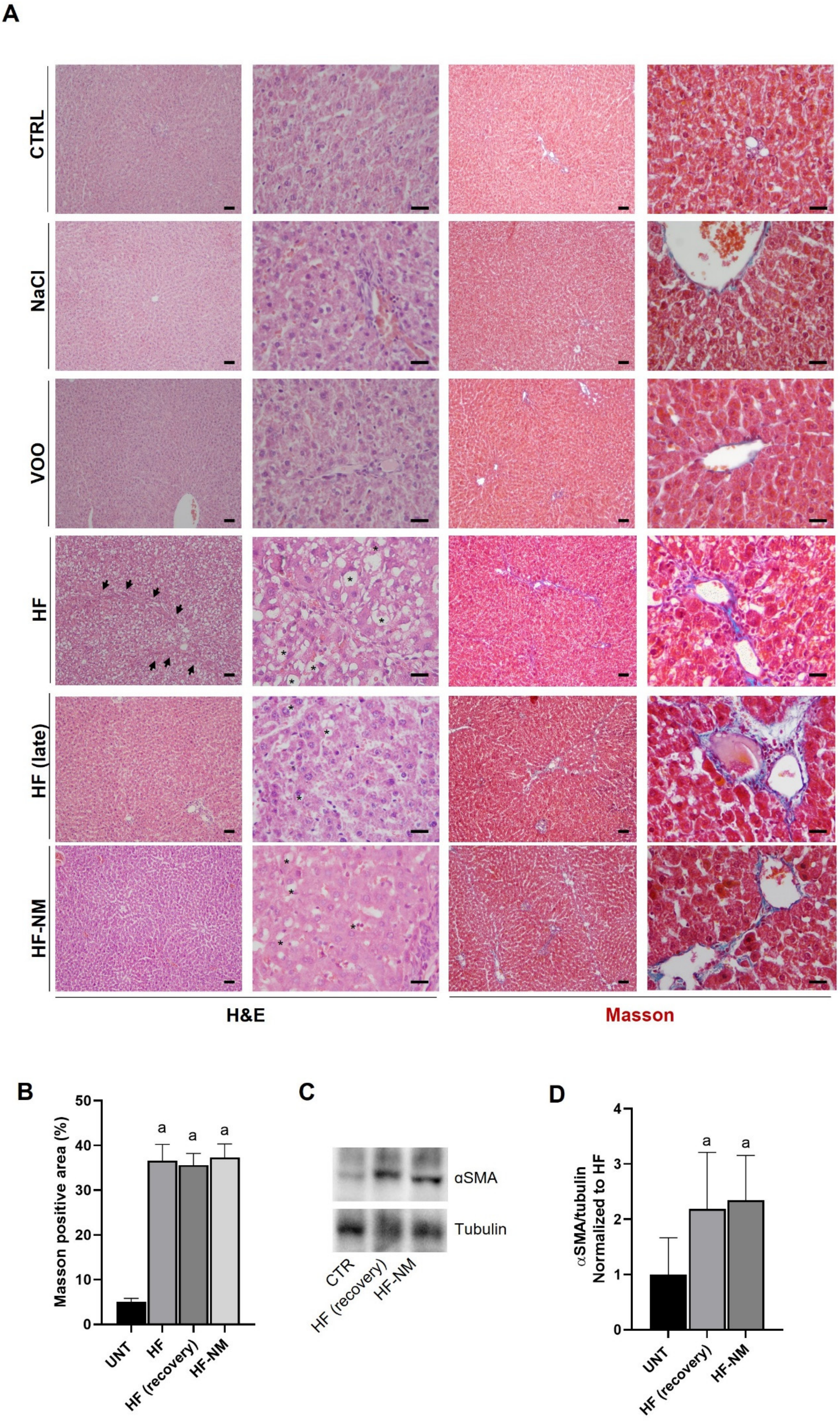
A
CTRL
NaCl
VOO
HF
HF (late)
HF-NM
H&E
Masson
B
Masson positive area (%)
50
40
30
20
10
0
a
a
a
UNT
HF
HF (recovery)
HF-NM
C
αSMA
Tubulin
CTR
HF (recovery)
HF-NM
D
αSMA/tubulin
Normalized to HF
4
3
2
1
0
a
a
UNT
HF (recovery)
HF-NM

Figure S1. (A) Hematoxylin and eosin and Masson's stainings. Liver sections from untreated rats; and treated with vectors; and CCl_4-induced HF and treated with MNs or recovering. (B) Masson's trichrome positive areas. (C) α-SMA western blot and (B) densitometry analysis. CTRL (Group I): Control untreated rats allowed free access to food and water. NaCl (Group II): Rats intraperitoneal injected with sterile 0.9% NaCl solution three times a week for two weeks. VOO (Group III): Rats intraperitoneal injected with sterile virgin oil three times a week for two weeks. HF (recovery) (Group VIII): HF rats were left to recover for two weeks after the last injection of CCl_4 with free access to food and water (control of immune system responses); HF-NM (Group XI): HF rats were intraperitoneal injected with empty micelles three times a week for two weeks after the last injection of CCl_4 (negative control of proposed therapeutic model). The scale bar corresponds to 10 µm. (B) Quantification of Masson's trichrome positive areas. The amount of interstitial fibrosis was measured by quantifying the collagenous fibrotic areas stained in blue/green in 10 random liver fields per section from 4 different rats for each group. Images were analysed by using multiphase image analysis ImageJ with a software version 1.49s. Values are expressed as mean ± SD (n= 8). Significative values were reported with respect to Group I CTRL, a = $p < 0.05$. (C) Representative western blot of α-SMA. (D) Western blot of α-SMA. Values are expressed as fold change respect to CTRL ± SD (n=8). Tubulin was used as the loading control and for band density normalization. Significative values were reported with respect to Group I CTRL, a = $p < 0.05$.

Table S1 Primers used for RT-qPCR reaction

Gene	Forward primer	Reverse primer
Hsp47	CAAGGGAGTGGTGGAGGTGA	GCCTTGTTCTTGTCGATGGC
ColIa1	GCGAAGGCAACAGTCGATTC	TTCGATGACTGTCTTGCCCC
Cpt1a	TCCAAGTTCTCTAGCCCTGAGA	CAAGGTGATAATGTCCATCATGGC
FASN	GGACTTGGGTGCCGATTACA	CGTACGATGGTCACCCTCAA
GADPH	ACCCCCAATGTATCCGTTGT	GCCTGCTTCACCACCTTCTT
